# Efficacy of Palbociclib and Endocrine Treatment in Heavily Pretreated Hormone Receptor-positive/HER2-negative Advanced Breast Cancer: Retrospective Multicenter Trial

**DOI:** 10.4274/balkanmedj.galenos.2020.2019.11.143

**Published:** 2020-02-28

**Authors:** Atakan Demir, Nil Molinas Mandel, Semra Paydas, Gökhan Demir, Özlem Er, Nazım Serdal Turhal, Sevil Bavbek, Yeşim Eralp, Pınar Mualla Saip, Emine Nilüfer Güler, Adnan Aydıner, Başak Oyan Uluç, Sadettin Kılıçkap, Necdet Üskent, Nuri Karadurmuş, Mehmet Ali Kaplan, Mustafa Teoman Yanmaz, Hacer Demir, Özkan Alan, Taner Korkmaz, Polat Olgun, Özlem Sönmez Uysal, Kadri Altundağ, Şeyda Gündüz, Meral Günaldı, Murat Sarı, İsmail Beypınar, Gül Başaran

**Affiliations:** 1 Department of Medical Oncology, Acıbadem University, İstanbul, Turkey; 2 Clinic of Oncology, American Hospital, İstanbul, Turkey; 3 Department of Medical Oncology, Çukurova University School of Medicine, Adana, Turkey; 4 Clinic of Oncology, Anadolu Medical Center Hospital, Kocaeli, Turkey; 5 Self-employed, İstanbul, Turkey; 6 Department of Medical Oncology, İstanbul University Oncology Institute, İstanbul, Turkey; 7 Department of Medical Oncology, Hacettepe University School of Medicine, Ankara, Turkey; 8 Clinic of Medical Oncology T.C. Ministry of Health Gülhane Training and Research Hospital, Ankara, Turkey; 9 Department of Medical Oncology, Dicle University School of Medicine, Diyarbakır, Turkey; 10 Şişli Memorial Hospital, İstanbul, Turkey; 11 Department of Medical Oncology Afyon Kocatepe University School of Medicine, Afyon, Turkey; 12 Tekirdağ State Hospital, Tekirdağ, Turkey; 13 Near East University Hospital, Lefkoşa, TRNC; 14 Self-employed, Ankara, Turkey; 15 Antalya Memorial Hospital, Antalya, Turkey; 16 Clinic of Medical Oncology, Florya Medical Park Hospital, İstanbul, Turkey

**Keywords:** Breast cancer, CDK4/6 inhibitors, palbociclib

## Abstract

**Background:**

The synthesis of CDK4/6 inhibitors with endocrine treatment in two series of treatment has been widely accepted as the standard for patients with estrogen receptor-positive metastatic breast cancer. In spite of this, the activity of CDK4/6 inhibitors in patients with metastatic breast cancer who have progressed despite receiving multiple lines of treatment is not well understood.

**Aims:**

To report the activity and safety of a CDK4/6 inhibitor (palbociclib) in patients in whom at least three lines of treatment for ER+ metastatic breast cancer had failed.

**Study Design:**

Multicenter retrospective observational cohort study.

**Methods:**

In this retrospective observational cohort study, we included 43 patients who received palbociclib after at least three lines of systemic treatment for ER+/HER2− metastatic breast cancer.

**Results:**

The median progression-free survival in our population was 7 months (25^th^-75^th^ percentile, 4-10), and the median overall survival was 11 months (25^th^-75^th^ percentile, 6-19). Although there were some adverse events, palbociclib was generally well tolerated, so dose reduction was needed for only six patients (14%).

**Conclusion:**

The efficacy of palbociclib among heavily treated hormone receptor-positive/HER2− patients with advanced breast cancer was acceptable in terms of clinical benefit, and it was generally well tolerated among this population.

Breast cancer (BC) is a major cancer in women. Despite the numerous therapeutic options that are available, it remains incurable, earning it the notorious reputation of being the second most common cause of cancer death in Western populations, following lung cancer (1). BC is a heterogeneous disease and has a variety of subgroups according to clinical, pathological, and molecular features (2).

Approximately 80% of all BC cases are estrogen receptor-positive (ER+)/human epidermal growth factor receptor 2 negative (HER2-). According to current guidelines, sequential endocrine therapy (ET) is the main treatment recommendation for premenopausal and postmenopausal women with ER+/HER2- stage 4 BC (except for extensive visceral involvement) (3,4). Resistance to ET (acquired or *de novo*) will develop in the vast majority of these cases during therapy (5). Cyclin-dependent kinases and mammalian target of rapamycin (mTOR) signaling pathways are the main mechanism of resistance to ET (6). Currently, the most commonly recommended therapeutic options to overcome ET resistance are mTOR inhibition (everolimus) and CDK4/6 inhibition (palbociclib, abemaciclib, and ribociclib) (7,8,9).

The combination of CDK4/6 inhibitors with ET in two series of treatment has been widely accepted as the standard treatment for patients with ER+ metastatic breast cancer (mBC) (10,11,12). However, the activity of CDK4/6 inhibitors in patients with mBC progressing after multiple treatment lines is not well known. In this multicenter retrospective study, we report the activity and safety of a CDK4/6 inhibitor (palbociclib) in patients for whom at least three lines of treatment for ER+ mBC had failed.

## MATERIALS AND METHODS

In this retrospective observational cohort study conducted between 14 September 2015 and 22 November 2019, we included 43 patients from different medical oncology clinics in Turkey. Patients received palbociclib after at least three lines of systemic treatment for ER+/HER2− mBC (confirmed at diagnosis or on the basis of a metastatic lesion). Patients using fulvestrant or aromatase inhibitors with palbociclib were included in the study. Baseline was defined as the date of the first dose of palbociclib. All patients provided written informed consent to participate in the study, and ethical approval was given by our institution’s ethics committee (12 September 2019; approval no.: 2019-14/34).

The main outcomes of this trial were overall survival (OS) and progress-free survival (PFS). OS was determined from the date of initiation of therapy until death or last visit date. PFS was the time window from the beginning of treatment to radiological progression, death, or last visit date. We also report the frequency of adverse events related to therapy (e.g., neutropenia, anemia, thrombocytopenia, diarrhea).

We included patients with mBC who progressed according to response evaluation criteria in solid tumors (RECİST) criteria despite at least three lines of standard therapy. Our exclusion criteria were as follows: radiotherapy, surgery, or systemic treatment within 2 weeks; past medical history of cardiovascular disease (arrythmias including atrial fibrillation, torsades de pointes, long or short QT interval, prior myocardial infarction, coronary artery bypass grafting, heart failure, and pulmonary embolism); hypersensitivity of palbociclib; and suicidal behavior.

### Statistical analysis

The data were analyzed using descriptive statistical methods. Continuous variables are given as mean ± standard deviation, and categorical variables are given as percentages. Kaplan-Meier curves were created to determine OS and PFS. IBM SPSS Statistics version 21 software (IBM, Armonk, NY, USA) was used to analyze the data and generate graphical content.

## RESULTS

The median age of patients was 51 years (25^th^-75^th^ percentile, 44-58). All patients were diagnosed with mBC, and 30 patients (69.7%) were initially in stages I-III. All patients were ER+, and five (11.6%) were ER+/progesterone receptor-negative. Most of the patients were pathologically classified as having invasive ductal carcinoma (n=41; 95.3%). All patients were given at least three lines of treatment for mBC, including chemotherapy and ET. For most of the patients, adjuvant ET was tamoxifen (n=19; 44.1%). Other combinations were as follows: letrozole (n=6; 13.9%) and anastrozole (n=3; 6.9%). Five patients (11.6%) had received both a steroidal and a nonsteroidal AI. Fulvestrant was used in 39.5% of patients (n=17) before palbociclib/AI. Twenty-one premenopausal women had undergone surgical or medical castration as part of combination hormone therapy. Only one patient had been treated with the exemestane/everolimus combination. Most of the patients (55%; n=23) had received sequential monochemotherapy with standard drugs, including taxanes, capecitabine, gemcitabine, and anthracyclines, whereas a small minority had received combination chemotherapy regimens such as capecitabine/docetaxel; fluorouracil, doxorubicin, and cyclophosphamide; doxorubicin/docetaxel; or a combination of both approaches. The baseline characteristics of patients in the study cohort are outlined in Table 1.

**Figure 1:**
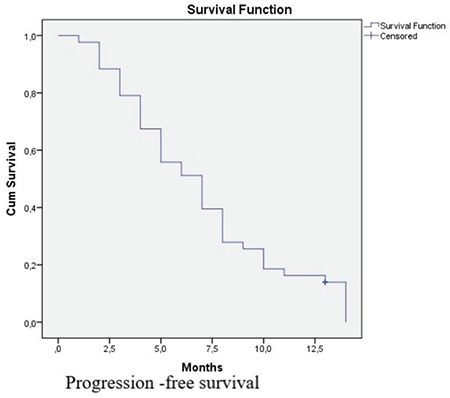
Overall Survival graphic for the whole group by Kaplan Meier curve [median OS: 11 months (25th-75th percentile, 6-19)].

**Figure 2:**
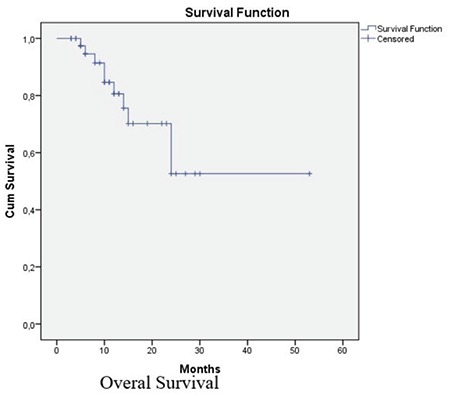
Progression-free Survival graphic for the whole group by Kaplan Meier curve [median PFS:7 months (25th-75th percentile, 4-10)].

According to RECİST criteria, no complete response was observed. Six patients (13.9%) had a partial response, 28 patients (65.1%) had stable disease, and 9 patients (21%) had progressive disease. The disease control rate was 79%, and the objective response rate was 13.9%. The median PFS in our population was 7 months (25^th^-75^th^ percentile, 4-10) (Figure 1), and the median OS was 11 months (25^th^-75^th^ percentile, 6-19) (Figure 2). All deaths in our study cohort were associated with disease progression.

Although there were some side effects, palbociclib was generally well tolerated. As a result, dose reduction was needed for only 6 patients (14%). The most common nonhematological side effect was nausea (n=30; 69.7%). Elevated transaminase levels were also frequent (n=25; 58.1%). Grade 3 neutropenia occurred in 12 patients (27.9%), and febrile neutropenia was seen in 2 patients (4%) (Table 2).

## DISCUSSION

CDK4/6 inhibitors are a critical step in the abolishment of ET resistance among ER+/HER2− patients. Many phase II and III clinical trials have been carried out to investigate the efficacy and safety of CDK4/6 inhibitors among this population (8,9,10,11). In the PALOMA-1 trial, the median PFS was 20.2 months in the letrozole/palbociclib arm and 10.2 months in the letrozole/placebo arm [hazard ratio, 0.488; 95% confidence interval (CI), 0.319-310.748; p=0.0004] (13). Similarly, in the PALOMA-2 trial, median PFS was 24.8 months in the palbociclib/letrozole arm and 14.5 months in the letrozole/placebo arm (hazard ratio, 0.58; 95% CI, 0.46-40.72; p<0.001) (14). The PALOMA-3 trial was performed to evaluate the efficacy of the combination of palbociclib and fulvestrant among 521 patients with hormone receptor-positive mBC as a second-line treatment option. The palbociclib-fulvestrant combination improved PFS significantly compared with the placebo-fulvestrant arm (9.2 vs 3.8 months; p<0.001) (9). Another CDK4/6 inhibitor, abemaciclib, was also tested in second-line treatment in the MONARCH 2 trial. The results were promising; the abemaciclib-fulvestrant combination prolonged PFS significantly (median PFS, 16.4 vs 9.3 months; p=0.001) (11).

In spite of CDK4/6 inhibitors their being well investigated among ER+/HER2− patients, there are no large phase 3 studies showing efficacy after treatment. Ban et al. (15) published a retrospective analysis of 24 heavily pretreated patients with ER+/HER2− mBC. All patients in their trial received a minimum of four lines of treatment for mBC, including chemotherapy and ET. They reported that 58.3% of patients achieved stable disease, and median PFS was 4.8 months and median OS was 11 months. They reported that grade III neutropenia occurred in 54.1% of patients (n=13), grade IV neutropenia in 12.5% of patients (n=3), and grade III thrombocytopenia occurred in 12.5% of patients (n=3). The most commonly reported treatment-related nonhematologic adverse event was nausea (12.5%; n=3). These side effects were consistent with our trial (15). In our trial, more patients achieved stability than in the trial by Ban et al. (15) (65.1% vs 58.3), and PFS was a bit longer in our trial (7 vs 4.8 months), which may be explained by the use different of treatment lines between trials (minimum three lines vs four lines), but the OS was similar in our study. A recent retrospective analysis also aimed to investigate the role of palbociclib among heavily treated (more than 4 previous CT lines) patients with mBC. The authors of that analysis reported the utility of palbociclib among 118 hormone receptor-positive/HER2− patients with advanced BC. The clinical benefit rate was 47.5%; overall response rate was 15.8%; median PFS was 4.5 months; and median OS was 15.8 months. In terms of therapeutic efficacy, PFS and response rate were comparable with our results, but our results were again favorable. Hematological side effects were consistent with our trial: 89.7% developed neutropenia (grade ≥3 in 56.8%), and 5.1% experienced febrile neutropenia. However, dose reductions and discontinuation rates were higher in the prior trial than in our trial; 48.3% had dose decreases after side effects, and 3.4% had palbociclib discontinued due to toxicity (16).

In summary, our trial adds important information to the literature about the use of palbociclib among highly treated hormone receptor-positive/HER2− patients with advanced BC. We demonstrated comparable PFS and OS rates among patients with advanced BC, and palbociclib was well tolerated except for febrile neutropenia among patients with such severe disease. The main limitation of our study is its retrospective nature and the relatively small number of patients. Additionally, we only determined PFS and OS, so we could not get any information about quality-of-life measures.

We demonstrated that the efficacy of palbociclib among heavily treated hormone receptor-positive/HER2− patients with advanced BC was comparable and generally well tolerated among this population. Further randomized controlled studies with a larger number of patients is needed to confirm our findings and define patients who may benefit even in late-stage disease.

## Figures and Tables

**Table 1:**
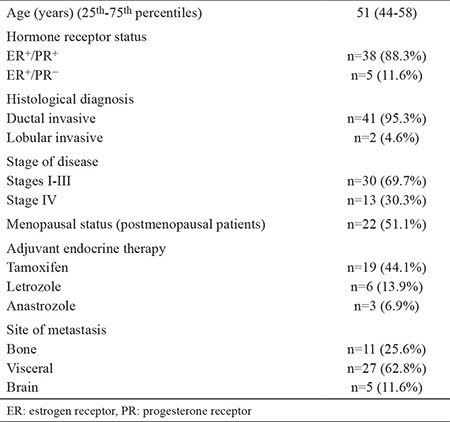
Baseline characteristics of patients in the study cohort

**Table 2:**
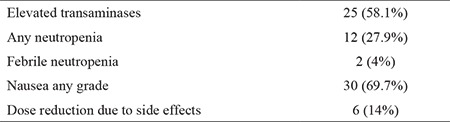
Adverse events during palbociclib treatment
